# Supplementary material for: Requirements of an Application to Monitor Diet, Physical Activity and Glucose Values in Patients with Type 2 Diabetes: The Diameter
Source: Nutrients. 2019 Feb 15;11(2):409. doi: 10.3390/nu11020409 (PMC6413029; doi:10.3390/nu11020409)
Supplement: Supplementary file 1 [file nutrients-11-00409-s001.pdf]

Supplementary

Table S1. Formulated requirements and why these requirements are important, as a result of the cohort study (Co), literature search (L), pilot study (P) and expert meetings (E) to be able to develop the dietary part of the Diameter. The requirements are formulated according to Function and events (F), Interaction and usability (I), Content and structure (C) and Style and aesthetics (S). The identification (ID) can be found in the first column, corresponding with the ID in the text. In the second column the priority level is shown as Must have (M), Should have (S), Could have (C) and Wish to have (W).

**Functions and events**				
ID	*Priority level*	*Origin*	*Requirement*	*Justification*
F1	M	Co	The registered food items in the past are used to learn often used products and meals to show smart options	Smart options based on the registration in the past decrease the number of actions, reducing the registration burden
F2	M	Co, P	The most frequently consumed products per individual are presented on top of each pre-defined meal moment (breakfast, lunch, dinner and in-between meals)	To decrease the number of actions taken to record commonly used food items
F3	M	Co	Dinner include pre-defined components: vegetables, pasta/rice/potatoes, meat/poultry/fish, gravy/sauce	To increase accuracy of the food record, pre-defined components will remember patients to complete their nutritional intake
F4	M	L, P	The food record measures and shows amount of carbohydrates	The amount of carbohydrates is of importance for patients with diabetes
F5	S	L, P	Show the effect of carbohydrates or specific food on the glucose values	This information is necessary for the patient to adequately act on the glucose changes and prevent hyperglycaemia.
F6	M	L, P	Calculate and give an overview of the amount of consumed calories, how many are left to eat that day and how many calories are burned	This insight is necessary for patients who want to lose of keep a stable weight.
F7	M	P	The time of ingestion is linked to the meal moments and the corresponding products with `now' or `other'	Time of ingestion is necessary to integrate the data of diet, activity and glucose values with each other
F8	M	P	Pre-defined household measures or portion sizes are linked to the products (e.g. 1 slice of bread is 35 gram)	To prevent that patients have to weigh or remember the amount of products every time something is consumed
F9	M	P	Registered products can be adjusted, added or removed	To ensure accuracy, patients can adapt their intake when not all food items were eaten or when other products were consumed
F10	M	P	The food record is divided in pre-defined meal moments: breakfast, lunch, dinner and in-between meals	In each category the meals can be entered in a way which is most logical for that meal moment (e.g. F1) to make the registration straightforward
F11	M	P	An overview can be given of the consumed calories, amount of fat, proteins and % dietary reference intake	Patients who want to lose weight or want to pay attention to the balance of ingestion need these insights on top of insight in carbohydrate intake (F4)
F12	W	P	A barcode scanner is integrated in the application	This proved a fast and easy way to recognize the right food product
F13	W	E	Photography technology is integrated in the application.	Useful for objective assessment and decreases the number of actions

F14	S	E	Registered food products should be recognized as a meal and saved to use it to give smart options in the future	When a part of the food products of a certain meal are inserted, a meal can be suggested based on previous entered meals. This can reduce the actions to register meals.
F15	C	E	Prior to the app use, specific information is asked for e.g. usage of butter on bread and additions in coffee and tea. This information is used to ask smart questions, e.g. when coffee is added the system asks 'did you add sugar and milk like you usually do?'	This is done to prevent that patients forget to add additions and to decrease the number of actions.
F16	S	E	Data output is stored per day and presented for breakfast, lunch, dinner, in-between meals and total carbohydrate intake	To gain insight in the carbohydrate distribution over the day for both the patient and diabetes nurse
F17	W	E	Patients can add new products to the database.	Products not included in the Dutch Food Composition Table should be added to ensure accuracy
**Interactions and usability**				
I1	S	Co	Show the (minimum) amount of days needed of completely registration per meal moment using count down	Knowing the amount of days will motivate the patients to register the asked days completely, knowing that in time the burden will decrease
I2	M	Co	The system asks if the patient drank something when food is entered without beverage	To prevent underreporting of nutrients caused by forgetting to enter drinks
I3	M	P	Suggestions are presented when typing in a food product	Registration of food will be faster when not the full name of the product needs to be entered.
I4	S	P	The system can be used on a laptop or PC	This makes it usable for a broader target population
I5	S	P	Notifications are sent when no food data has been entered	To stimulate input
I6	C	E	Own recipes/dishes can be entered to remember for future use	To reduce the need to register everything in detail in time
I7	M	E	The system can be used, independent of internet connection.	At any time of the day, patients should have access to the food record, to limit memory bias.
**Content and structure**				
C1	S	L	Give healthy recipe ideas	Make it easier for patients to choose healthy options
C2	M	L	Native language of the patient is used	In order to make sure the patient understands every provided information/part
C3	M	P	The Dutch Food Composition Table is used as nutritional database	The Dutch Food Composition Table features nutritional values of 2300 mainly Dutch products and is the most elaborated food database in the Netherland which can be used to register food consumption with their corresponding nutritional values
C4	M	P	All possible names which can be used for a single product are connected to that product	This is necessary to easily find the right food product
C5	M	P	Information regarding food and healthy choices is given	To increase the knowledge of patients of different food products and nutrients
C6	W	E	Give alternative healthier option of a product the patient entered (e.g. low	Based on this information patients potentially will make healthier choices in the future

			carbohydrate bread instead of normal bread).	
C7	M	E	A guideline is available with instructions to record food.	Detailed instructions of recording food will improve self-management
C8	M	E	Data output is presented in gram per day.	For each product the amount of e.g. carbohydrates in gram per day should be calculated, based on nutritional values per 100 gram of the food item as noted in the Dutch Food Composition Table.
**Style and aesthetics**				
S1	M	P	Time has to be entered before a meal or food product can be registered	This prevents that patients ignore the time field and makes sure the data of food, activity and glucose can be integrated
S2	M	P	A standard display of the current date is linked to each new food record, but this date can be manually changed	Decreases the number of actions when reporting current food items
S3	W	P	Pictures of the food products are shown	Pictures make it easier to choose the right product out of a list
S4	C	P	Information is given as a short fact which changes every day	To increase the knowledge of a patient step-by-step in an attractive way
S5	W	E	Drag pictures of food to a plate	This makes it easier to estimate the portion size of dinner and reduces the need of typing and reading text

Table S2. Formulated requirements and why these requirements are important, as a result of the cohort study (Co), literature search (L), pilot study (P) and expert meetings (E) to be able to develop the physical activity and sedentary behaviour part of the Diameter. The requirements are formulated according to Function and events (F), Interaction and usability (I), Content and structure (C) and Style and aesthetics (S). The identification (ID) can be found in the first column, corresponding with the ID in the text. In the second column the priority level is shown as Must have (M), Should have (S), Could have (C) and Wish to have (W).

**Functions and events**				
ID	*Priority level*	*Origin*	*Requirement*	*Justification*
F18	C	Co	Giving notification when the battery of the activity tracker is almost empty	To prevent data loss the battery of the sensor needs to be charged on time
F19	M	Co	Reminder to wear the sensor when it is detected that the sensor has no data for a longer period	To prevent data loss the patient has to wear the activity sensor
F20	W	Co	Automatically detect activities which can not be detected by the activity tracker like bicycling	Activities like bicycling and swimming can not be measured properly using the Fitbit, but are important to detect
F21	S	Co	Detect maximal intensity of MVPA or activity bout of an individual patient	MVPA differs per patient and is dependent on different factors. It should be possible to examine this.
F22	S	E	Start with a baseline week before insight is given to the patient.	This needs to be done to evaluate what the starting value is of an individual patient
**Interactions and usability**				
I8	M	Co	Manual insertion of activities which can not be detected by the activity tracker	Activities like bicycling and swimming can not be measured properly using the Fitbit, but are important to detect
I9	S	L	Give notification to give a reminder to move	To prevent not enough movement during the day

I10	S	L	Weight can be manually registered	To show to weight (changes) in time
I11	C	L	Detect periods of sedentary behaviour	To be able to develop the coaching part in the future, sedentary periods needs to be detected to be able to break these periods.
I12	W	E	Multiple types of activity trackers can be used	Making it usable for a broad target group without the need to buy a specific sensor
**Content and structure**				
C9	C	Co, P	Give information concerning activity bouts and physical activity	The patients need motivation to be more active, therefore they need insight why this is important
C10	C	Co, L, P	Giving patients information to increase knowledge concerning sedentary behaviour	To reduce long periods of sedentary behaviour
C11	C	L	Show the burned calories during exercise	This insight can help patient who want to prevent increase of weight
C12	C	L	Show the progress of weight over time	To give the insight if the weight changes
**Style and aesthetics**				
S6	S	L	Show activity visually using bar charts	This clearly shows in one overview if the goal of that day has achieved.

Table S3. Formulated requirements and why these requirements are important, as a result of the cohort study (Co), literature search (L), pilot study (P) and expert meetings (E) to be able to develop the glucose part of the Diameter. The requirements are formulated according to Function and events (F), Interaction and usability (I), Content and structure (C) and Style and aesthetics (S). The identification (ID) can be found in the first column, corresponding with the ID in the text. In the second column the priority level is shown as Must have (M), Should have (S), Could have (C) and Wish to have (W).

**Functions and events**				
ID	*Priority level*	*Origin*	*Requirement*	*Justification*
F23	M	Co, L, P	Display the glucose values, including the percentage of hypo-, hyper- and normoglycaemic events, throughout the day and the progression over time	This gives the insight to become better able to keep the values between the target ranges
F24	M	Co	Display the glucose variability during the day	This insight is necessary for the patients so they are able to manage their glucose values
F25	M	E	Blood glucose value thresholds are adjustable	The thresholds can differ per patient
F26	W	E	Can synchronize with different glucose monitoring devices and insulin	This makes it usable for a broader target population
**Interactions and usability**				
I13	M	Co	Give notification to scan the sensor	To reduce data loss
I14	M	Co	Be able to connect a new Freestyle Libre sensor	The maximum days one sensor can be used is 2 weeks
I15	M	L	Wirelessly transferring of the glucose data	This makes it easier to use the sensor and the application in daily living
I16	W	P	Give a warning when blood glucose levels are higher or lower than predefined thresholds	This can help the patient to act on hyper, or hypoglycaemic episodes
I17	M	E	Measured glucose values by a finger-prick can manually be inserted	Some patients do not use CGM, in this case finger-prick data needs to be gathered. Also, this data can be used to compare this data with CGM data for research
**Content and structure**				

C13	M	Co, L, P	Give information about glucose values, glucose targets, glucose variability and overall diabetes	To give patients knowledge to better manage their glucose values and diabetes
C14	C	E	Predict glucose values based on the data	To give patients the option to act on time on changes of their glucose values
**Style and aesthetics**				
S7	S	L	Show the glucose values and how activity and nutrients correlate or affect the blood glucose in an easy-to-understand way	Giving the patients the insight to optimal manage their diabetes

Table S4. Formulated requirements and why these requirements are important, as a result of the cohort study (Co), literature search (L), pilot study (P) and expert meetings (E) for the shared part of the Diameter. The requirements are formulated according to Function and events (F), Interaction and usability (I), Content and structure (C) and Style and aesthetics (S). The identification (ID) can be found in the first column, corresponding with the ID in the text. In the second column the priority level is shown as Must have (M), Should have (S), Could have (C) and Wish to have (W).

**Functions and events**				
ID	*Priority level*	*Origin*	*Requirement*	*Justification*
F27	C	P	Based on all the data the device calculates the insulin bolus	Making it easier and more precise for patients to calculate the insulin they need based on the data
F28	M	E	The data should be stored according to the European privacy laws.	To prevent privacy problems, the data must be stored following the European privacy laws
F29	M	E	The times of all different sensors are synchronized	To ensure every device uses the same time
**Interactions and usability**				
I18	S	L	A connection with a (digital) healthcare professional is available	If the care professional has insight in the gathered data, he can help the patient with an optimal diabetes treatment
I19	M	L	Data output provides an overview of the carbohydrate intake, activity, glucose values and the variability of these three for to the care professional	The care professional needs enough information to tailor the treatment plan and the data needs to be collected over enough time so progress can be visible
I20	S	L	The time spent to actively use the application is less than 15 minutes a day	To prevent patients to stop using the application
I21	M	L	The app is provided for free	Most people are unwilling to pay anything for apps and discontinuing use when they find that in-app payments are required
I22	S	P	Blood pressure measurements can be registered	A good blood pressure management is for a part of the patients important, it is desired that this can also be done using the app, to prevent the need of other applications
I23	S	E	Have an option to choose on which parts information and education is desired	In this way patient can focus on one part instead of everything all together which can give information overload
I24	M	L	The patient is able to enter the desired data without assistance	Everyone meeting the inclusion criteria should be able to use all parts of the app, independent of education level
**Content and structure**				
C15	M	L	The language and tone are accessible, encouraging and supportive	To keep patients wanting to use the application

C16	M	L	Terminology is used where needed, but definitions and explanations are provided	Using the medical terminology with an option to show the explanation makes it attractive for as well highly educated as low educated patients
C17	M	L	Native language of the patient is used	In order to make sure the patient understands every provided information/part
C28	M	E	Data can be stored at least 1 year.	The patient and practitioner need stored data to get insight in the nutritional intake and trends over time
**Style and aesthetics**				
S8	M	L	Generally intelligible symbols and terms are used	This makes it more attractive and easier to use the app
S9	M	L	Scrolling is minimized	Scrolling makes it less clear and takes time, this does not encourage usage of the app
S10	S	E	An overview of the data in the past can be found showed in an overview per day, per week and per month	To see the course in time
